# Regulator of G Protein Signaling Proteins Control Growth, Development and Cellulase Production in *Neurospora crassa*

**DOI:** 10.3390/jof8101076

**Published:** 2022-10-13

**Authors:** Ilva E. Cabrera, Yagna Oza, Alexander J. Carrillo, Logan A. Collier, Sara J. Wright, Liande Li, Katherine A. Borkovich

**Affiliations:** Department of Microbiology and Plant Pathology, University of California-Riverside, 900 University Avenue, Riverside, CA 92521, USA

**Keywords:** filamentous fungi, heterotrimeric G protein signaling, genetic epistasis, regulator of G protein signaling, cellulase activity, Neurospora

## Abstract

Heterotrimeric (αβγ) G protein signaling pathways are critical environmental sensing systems found in eukaryotic cells. Exchange of GDP for GTP on the Gα subunit leads to its activation. In contrast, GTP hydrolysis on the Gα is accelerated by Regulator of G protein Signaling (RGS) proteins, resulting in a return to the GDP-bound, inactive state. Here, we analyzed growth, development and extracellular cellulase production in strains with knockout mutations in the seven identified RGS genes (*rgs-1* to *rgs-7*) in the filamentous fungus, *Neurospora crassa.* We compared phenotypes to those of strains with either knockout mutations or expressing predicted constitutively activated, GTPase-deficient alleles for each of the three Gα subunit genes (*gna-1*^Q204L^, *gna-2*^Q205L^ or *gna-3*^Q208L^). Our data revealed that six RGS mutants have taller aerial hyphae than wild type and all seven mutants exhibit reduced asexual sporulation, phenotypes shared with strains expressing the *gna-1*^Q204L^ or *gna-3*^Q208L^ allele. In contrast, Δ*rgs-1* and Δ*rgs-3* were the only RGS mutants with a slower growth rate phenotype, a defect in common with *gna-1*^Q204L^ strains. With respect to female sexual development, Δ*rgs-1* possessed defects most similar to *gna-3*^Q208L^ strains, while those of Δ*rgs-2* mutants resembled strains expressing the *gna-1*^Q204L^ allele. Finally, we observed that four of the seven RGS mutants had significantly different extracellular cellulase levels relative to wild type. Of interest, the Δ*rgs-2* mutant had no detectable activity, similar to the *gna-3*^Q208L^ strain. In contrast, the Δ*rgs-1* and Δ*rgs-4* mutants and *gna-1*^Q204L^ and *gna-2*^Q205L^ strains exhibited significantly higher cellulase activity than wild type. With the exception of sexual development, our results demonstrate the greatest number of genetic interactions between *rgs-1* and *gna-1* and *rgs-2* and *gna-3* in *N. crassa*.

## 1. Introduction

The filamentous fungus *Neurospora crassa* colonizes new environments through the polar growth, branching and fusion of tube-like structures called hyphae to form a networked structure, the mycelium [1,2,3]. As hyphae grow, crosswalls (septa) are laid down between cell compartments [4]. Septa have pores that allow movement of small molecules, proteins and even organelles, facilitating cell–cell communication throughout the mycelial colony [5].

*N. crassa* uses three different developmental pathways to produce spores for dispersal [1,6,7]. The major asexual sporulation pathway, macroconidiation, results in production of multinucleated spores (macroconidia) [6]. These macroconidia develop via a budding routine from the tips of aerial hyphae and are easily released into the environment upon maturation. Spores produced during the second asexual sporulation pathway, microconidia, contain only one nucleus and develop from basal hyphae [8]. The third type of spore, the ascospore, is produced during sexual reproduction between male and female cells of opposite mating type [7]. Fertilization is accomplished when female reproductive structures (protoperithecia) extend chemotropic hyphae (trichogynes) towards a microconidium, macroconidium or hyphal fragment (male) from a strain of opposite mating type. The meiotic products (ascospores) are ejected from the mature fruiting body (perithecium) into the environment and are able to germinate to form a colony upon activation by heat, such as during a fire [7].

Previous studies have demonstrated that perturbation of components in the G protein signaling pathway results in altered growth and development in *N. crassa* [9]. *N. crassa* possesses three Gα proteins GNA-1, GNA-2, and GNA-3 (Guanine Nucleotide-binding protein Alpha—1/2/3) [10,11,12,13], two predicted Gβ proteins GNB-1 (Guanine Nucleotide-binding protein Beta -1) and CPC-2 (Cross Pathway Control -2) [14,15,16] and one Gγ protein GNG-1 (Guanine Nucleotide-binding protein Gamma -1) [17]. Various G protein subunits have been shown to regulate hyphal growth rate, aerial hyphae height, macroconidia abundance, development of protoperithecia and perithecia, and stress resistance. Alterations in adenylyl cyclase activity and protein levels and the resulting effects on cAMP amount have been identified as downstream effects of G protein signaling in *N. crassa* [13,18,19,20]. Our group has recently demonstrated that five of the six predicted G protein subunit genes (including the Gα subunits *gna-1* and *gna-3*) and adenylyl cyclase are required for production of detectable cellulase activity in *N. crassa* [21].

Regulator of G protein Signaling (RGS) proteins have been demonstrated to function as negative regulators of G protein pathways in numerous eukaryotes [22,23,24,25]. RGS proteins act as GTPase Activating Proteins (GAPs) by accelerating hydrolysis of GTP bound to Gα subunits by more than 2000 times, returning the Gα more rapidly to the inactive GDP-bound state [23]. Functions for certain RGS proteins have been characterized in several fungal systems, including the ascomycetes *Saccharomyces cerevisiae*, *Aspergillus nidulans*, *Aspergillus fumigatus*, *Aspergillus flavus*, *Fusarium verticillioides* and *Magnaporthe oryzae* [26,27,28,29,30,31]. A recent study provided evidence that an RGS protein (RGS-1) negatively regulates the alternative oxidase gene *aod-1* in *N. crassa* [32]. However, functions for RGS-1 and the other RGS proteins in regulation of growth and development have not yet been studied in *N. crassa.* Furthermore, roles for RGS proteins in cellulase production have not been systematically analyzed in any fungal system.

In this study, we characterize the phenotypes of mutants lacking each of the seven predicted RGS genes in *N. crassa*. We compare RGS mutant defects to those of strains carrying null or constitutively activated, GTPase-deficient mutations in the three Gα genes. Our results implicate genetic interactions between particular Gα-RGS partners during regulation of growth, asexual and sexual development, and extracellular cellulase production in *N. crassa*.

## 2. Materials and Methods

### 2.1. Media, Strains and Genetic Procedures

Vogel’s minimal medium (VM) [33] was used for strain propagation and assays of vegetative growth and development, with the exception that the carbon source was either (all expressed as wt/vol) 1.5% sucrose (normal concentration), 2% glucose or 2% crystalline cellulose (Avicel-PH101, 50-µm particle; Sigma-Aldrich, St. Louis, MO, USA), as indicated in the Results and Figure Legends. Sorbose-containing medium (FGS) was used to facilitate colony formation on plates [34]. Where indicated, media contained 100 μg/mL histidine, 200 μg/mL hygromycin B (Calbiochem, San Diego, CA, USA), 200 μg/mL nourseothricin (for *nat*^+^ selection; [35] (Werner BioAgents, Jena, Germany), or 400 μg/mL phosphinothricin purified from Finale (Bayer Crop Science, St. Louis, MO, USA), for *bar*^+^ selection, as described previously [36]. Medium used for *bar*^+^ selection contained proline as the nitrogen source [37]. Macroconidia from 5- to 7-day-old VM agar flasks or slant cultures or packed hyphae were used to inoculate all media, as previously described [16]. Submerged liquid cultures were inoculated at a density of 1 × 10^6^ macroconidia/mL and incubated for 16 h at 30 °C with shaking at 200 RPM. *Escherichia coli* strain DH5α was used to maintain plasmids.

*N. crassa* strains used in this study are listed in Table 1. All RGS gene deletion mutants were constructed by the *Neurospora* genome project using homologous recombination at the RGS gene locus, replacing the gene with a hygromycin B resistance cassette as described [38]. The Δ*rgs-3*, Δ*rgs-5*, Δ*rgs-6* and Δ*rgs-7* mutants were obtained as homokaryons, while the Δ*rgs-1*, Δ*rgs-2*, and Δ*rgs-4* mutants were obtained as heterokaryons, all in the *mat a* mating type. Homokaryons in both the *mat a* and *mat A* mating types were obtained by crossing heterokaryotic strains as males to wild-type strain 74A (Table 1; FGSC 987, *mat A*) using standard methods, with selection of progeny on sorbose plates [34] supplemented with hygromycin B. Genomic DNA was isolated from progeny and strain genotypes were verified by Southern analysis using the knockout cassette as a probe [38] or by diagnostic PCR using pairs of gene-specific and *hph* cassette-specific primers (Table S1; Figure S1).

Double mutants lacking an RGS gene and the Δ*mus-52*::*nat*^+^ mutation were produced using sexual crosses between single mutants, with the first selection on medium containing hygromycin. Use of the Δ*mus-52*::*bar*^+^ background results in a high frequency of homologous recombination in *N. crassa* [38] and was necessary for proper targeting of the RGS complementation constructs to the *pan-2* locus (see below). Hygromycin-resistant progeny were spot-tested on medium containing nourseothricin, and nourseothricin-resistant strains selected for further analysis. DNA was isolated from progeny and diagnostic PCR performed as described above. Strains carrying both mutations were carried forward (Table 1).

Complementation constructs were made that would allow expression of each RGS gene *in trans* to the Δ*rgs* knockout mutation. The vector backbone was pccg-1MCSV5bar-2, which targets DNA to (and deletes) the *pan-2* locus, leading to pantothenate auxotrophy and resistance to phosphinothricin. pccg-1MCSV5bar-2 is based on pRS426PVG [39] and contains in order, the 1kb region 5′ to the *pan-2* open reading frame (ORF), the *ccg-1* promoter amplified from pMF272 [40], a multiple cloning site, 5xGlycine linker, V5 epitope tag, the *bar* gene conferring resistance to phosphinothricin, and the 1kb region 3′ to the *pan-2* ORF. pccg-1MCSV5bar-2 is also a yeast-*E. coli* shuttle vector that confers uracil prototrophy to *S. cerevisiae ura3* mutants and ampicillin resistance in *E. coli*. Primers were used to amplify the ORF of each RGS gene from *N. crassa* genomic DNA using PCR (Table S1). pccg-1MCSV5bar-2 was digested with *Pac*I and transformed along with each amplified RGS ORF fragment into yeast strain FY834 to facilitate yeast recombinational cloning, as previously described [38]. The complementation constructs were transformed using electroporation into the appropriate Δ*rgs*, Δ*mus-52::bar*^+^ strain (Table 1). Transformants were plated on medium containing phosphinothricin and pantothenate. Genomic DNA was checked for replacement of the *pan-2* gene with the RGS ORF using diagnostic PCR (Primers in Table S1; Figure S2). Selected strains used for experiments are noted in Table 1.

### 2.2. Growth and Developmental Phenotypic Assays

Phenotypic assays for basal hyphae growth rate, aerial hyphae height and macroconidia abundance were conducted as described [16]. Wild type strains (Table 1; FGSC 4200, *mat a* and FGSC 2489, *mat A*) were used as controls. Four biological replicates were used for basal hyphae growth rate calculations, six were used for quantitation of macroconidia amount and 12 were used to determine aerial hyphae height. For sexual development assays, strains were inoculated on slants containing synthetic crossing medium agar (SCM); [41] and incubated in constant light for one week at room temperature. Development of female reproductive structures (protoperithecia) was scored at 7 days after inoculation. Cultures were then fertilized using macroconidia from a wild type strain of opposite mating type and incubation continued under the same conditions. Formation of perithecia was scored one week after fertilization and ascospore ejection two weeks after fertilization.

### 2.3. Assessment of Gene Expression, Growth, Supernatant Protein, Cell Mass Protein and Cellulase Activity Measurements with Avicel as the Carbon Source

RNAseq data were obtained from [42] and used to extract expression levels for the Gα and RGS genes in wild type during growth on sucrose vs. Avicel as a carbon source. For visual inspection of growth in Avicel, macroconidia were inoculated into 25 mL VM-Avicel liquid cultures at a concentration of 1 × 10^6^ cells/mL. Cultures were grown at 25 °C in constant light with shaking at 200 RPM for 3 days. Cultures were collected and centrifuged as described [21]. The pellets containing hyphal mats and residual Avicel were photographed.

For measurement of cellulase activity, strains were cultured in VM-Avicel as the carbon source as described above. Cell-free culture filtrates were obtained by passing culture supernatants through a 0.45 μm filter. The filtrate was used for assay of glucose release (cellulase) activity from Avicel as described in [21], using a coupled enzyme assay. Protein was extracted from the cell pad using sodium dodecyl sulfate and heat as described [21]. The protein concentration in the cell pad extracts and cell-free supernatants was quantitated using the Pierce Bicinchoninic acid (BCA) protein assay (Thermo Fisher Scientific, Chino, CA, USA), with bovine serum albumin (BSA) as described in [21]. Cellulase activity was normalized to biomass protein. The final glucose release cellulase activity is expressed as nmole glucose/mL supernatant/mg of extracted biomass protein. For visualization of protein species in the samples, the cell-free supernatants were concentrated five-fold using centrifugal filter units and then equal volumes electrophoresed on 10% SDS-PAGE gels as described [21]. Gels were stained using Coomassie Brilliant Blue as described [13].

### 2.4. Statistical Analysis

Grubb’s Q test was utilized to detect and eliminate outliers for all studies [43]. For quantitative growth and developmental assays (basal hyphae growth rate, aerial hyphae height and macroconidia abundance), pairwise Student’s *t*-tests [44] were performed using base R with no pooling of standard deviations. Each comparison was an unpaired *t*-test and the *p*-values generated were adjusted using the Holm-Bonferroni method [45]. Bar graphs were made using ggplot2 [46] or Microsoft Excel (Microsoft, Redmond, WA, USA). For analysis of supernatant protein, cell mass protein and cellulase activity after growth in Avicel, bar graphs were created using Microsoft Excel and Student’s *t*-test [44] (paired, two-tailed) was performed using Microsoft Excel. For figures, *p*-value significance levels are denoted as * *p* < 0.05; ** *p* < 0.01, and *** *p* < 0.001.

## 3. Results

### 3.1. The N. crassa Genome Contains Seven Predicted RGS Genes

**Figure 1:**
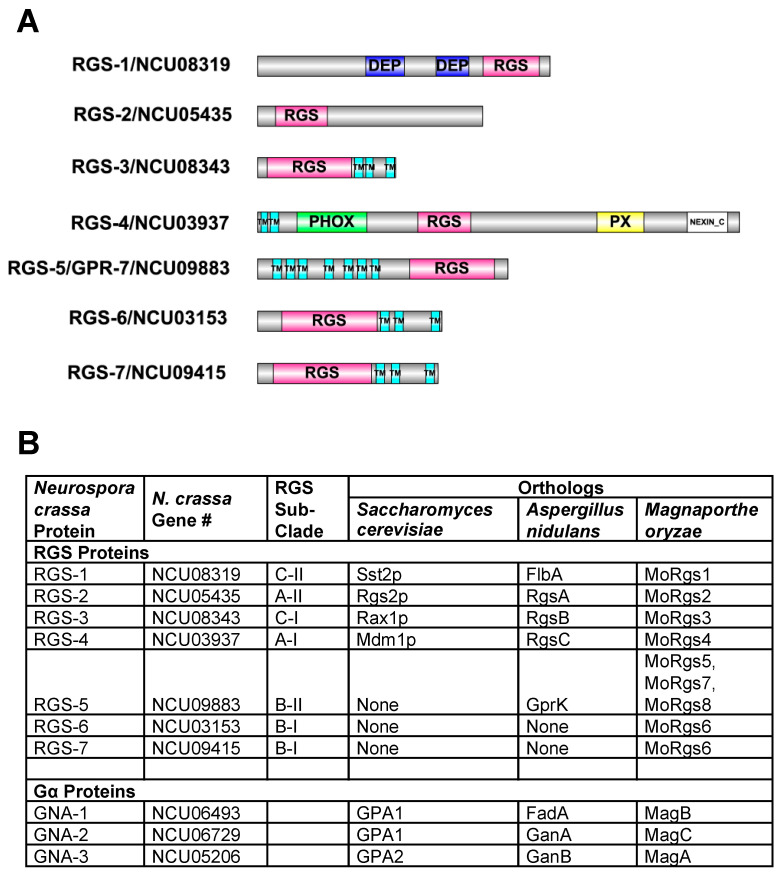
RGS protein domain structures and orthologs in other fungi. (**A**) Protein domains of the seven RGS proteins. Gene names and numbers are shown to the left of each protein cartoon. The protein domains in each RGS protein were determined using Interpro. Domains: RGS = Regulator of G protein Signaling domain; DEP = Dishevelled, Egl-10 and Pleckstrin domain; PX = Phosphoinositide binding domain; PHOX = Phagocytic Oxidase domain; TM = Transmembrane domain; NEXIN_C = Sorting Nexin C-terminal domain; (**B**) RGS and Gα protein orthologs in selected fungi. Features of fungal orthologs of the seven RGS and three Gα proteins from *N. crassa* are presented. RGS subclades are taken from reference [26].

RGS genes have been identified in numerous fungal species, including *Saccharomyces cerevisiae*, *Aspergillus nidulans* and *Magnaporthe oryzae* (rev. in [26]). BLAST searches of the *N. crassa* genome sequence revealed evidence for seven genes encoding RGS proteins [26]; (this study). All seven *N. crassa* RGS proteins contain a highly conserved RGS box [47] (Figure 1A). RGS-3, RGS-4, RGS-6 and RGS-7 possess 2-3 transmembrane domains. RGS-5 contains seven transmembrane helices, similar to G Protein Coupled Receptors, and was previously characterized as GPR-7 in *N. crassa* [48]. RGS-1 possesses two Dishevelled/Egl-10/Pleckstrin (DEP) domains, implicated in membrane association and found in many proteins involved in spatial regulation of signal transduction [49]. In addition to its two transmembrane helices, RGS-4 contains PX, PHOX and NEXIN_C domains. These three domains are typically found on proteins involved in protein sorting/secretion. PX and PHOX mediate phosphoinositide binding [50,51], while the NEXIN_C (sorting Nexin, C-terminal) motif is found on proteins involved in endosomal sorting [52].

Using phylogenetic analysis, three major clades of RGS proteins were identified in fungi, and these were further divided into six sub-clades, termed A-I, A-II, B-I, B-II, C-I and C-II (Figure 1B) [26]. Using this organizational framework [26], we note that *N. crassa* has at least one ortholog in all six sub-clades, with two in the B-I class (RGS-6 and RGS-7). In contrast, *S. cerevisiae* has only four of the sub-clades and *A. nidulans* lacks the sub-clade with two *N. crassa* members (B-I; Figure 1B). *M. oryzae* has at least one member in the six sub-clades, with three in B-II ([26]; Figure 1B; although *M. oryzae* RGS-5 lacks transmembrane domains). In spite of the observation that *N. crassa* RGS-3, RGS-6 and RGS-7 have a similar protein domain structure (Figure 1A), RGS-3 is well-separated in a sub-clade distinct from RGS-6 and RGS-7 in the phylogenetic tree [26]. Regarding Gα subunits, *N. crassa*, *A. nidulans* and *M. oryzae* all share three conserved proteins, while the yeast *S. cerevisiae* has two Gα proteins (Figure 1B).

### 3.2. Assessment of Quantitative Phenotypes in Strains with Mutations in RGS or Gα Subunit Genes during Growth and Asexual Development

Previous research has shown that the three Gα protein subunits have important functions during growth and development in *N. crassa*, with GNA-2 often serving a compensatory role to GNA-1 and GNA-3 [11,12,13,19,20]. In this study, we investigated roles for the seven RGS genes during the *N. crassa* life cycle. We analyzed single mutants lacking an RGS or Gα gene and strains expressing a constitutively active, GTPase-deficient Gα allele (*gna-1*^Q204L^, *gna-2*^Q205L^ or *gna-3*^Q208L^). Since RGS proteins function as negative regulators of Gα proteins, we expect that if a particular RGS regulates a specific Gα subunit, we would observe similar phenotypes in strains deleted for the RGS gene and those that express a constitutively active Gα gene allele. In order to ensure that phenotypes in the *rgs* mutants resulted from loss of the *rgs* gene, we also analyzed *rgs* deletion mutants expressing a *pan-2* targeted version of the RGS gene *in trans* (see Section 2 and Table 1). These complemented strains exhibited significant or complete complementation of phenotypes during the lifecycle, including hyphal growth rate, aerial hyphae height, macroconidiation, and female fertility during sexual development (Figure S3).

**Figure 2:**
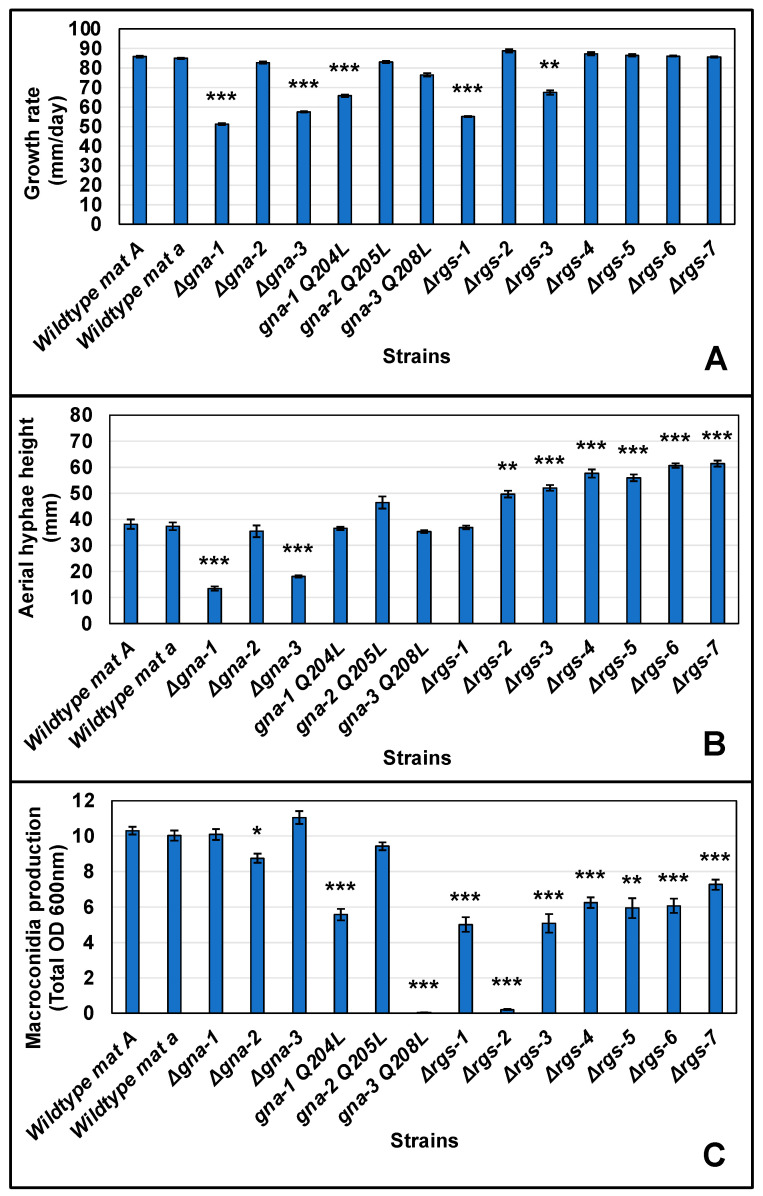
Quantitative phenotypes during growth and development. (**A**) Growth rate of basal hyphae. VM agar race tubes with sucrose as the carbon source (VM-sucrose) were inoculated with the indicated strains (see Table 1 for genotypes) and then incubated at 25 °C in constant darkness. Linear growth rates were determined using at least four biological replicates. Error was calculated as the Standard Error. A pair-wise Student’s *t*-test was performed between all strains (See Figure S4), but this graph only shows comparisons to the wildtype *mat A* strain. *p*-value thresholds are indicated as * *p* < 0.05, ** *p* < 0.01, and *** *p* < 0.001; (**B**) Aerial hyphae height measurements. Culture tubes containing liquid VM-sucrose medium were inoculated with the indicated strains and then incubated statically in constant darkness for three days at room temperature. The distance grown by aerial hyphae above the liquid interface was then measured in mm. Values are the average of 12 replicates. Error calculations and Student’s *t*-test were performed as described in (**A**). A summary of all pair-wise comparisons can be found in Figure S5; (**C**) Macroconidia production. Slant tubes containing VM-sucrose agar medium were inoculated with the indicated strains and then cultured for three days at 30 °C in constant darkness, followed by four days in constant light at room temperature. Macroconidia were harvested from the cultures using water and filtered through Handiwipes™ to remove basal and aerial hyphae. Macroconidia in the filtrate were then pelleted using centrifugation and brought to a known volume using water. The OD600 nm (proportional to macroconidia/mL) was determined for 1 mL of suspension and used to calculate total OD600 nm. Values represent six biological replicates. Error calculations and Student’s *t*-test were performed as described in (**A**). A summary of all pair-wise comparisons can be found in Figure S6.

We first assessed the effects of mutations in the RGS and Gα genes on the growth rate of basal hyphae when *N. crassa* is cultured on solid medium (Figure 2A and Figure S4). Consistent with results from previous studies, of the three Gα subunit mutants, those lacking *gna-1* or *gna-3* had significantly slower growth rates relative to wild type [11,13]. Strains carrying the *gna-1*^Q204L^ allele also had significantly slower growth rates than wild type *mat A*. Of the RGS mutants, Δ*rgs-1* and Δ*rgs-3* strains were significantly slower than wild type (~64% of wild type *mat A*). Based on these results, it is plausible that *rgs-1* and/or *rgs-3* have a genetic interaction with *gna-1*, as the *gna-1*^Q204L^ and Δ*rgs-1* and Δ*rgs-3* strains all grow more slowly than wild type. In particular, the *gna-1*^Q204L^ and Δ*rgs-3* strains are indistinguishable from one another (Figure S4). However, this interpretation is complicated by the observation of slower growth in strains with null or activating mutations in *gna-1*.

We next explored phenotypes during asexual sporulation (macroconidiation). Regarding the height of aerial hyphae, six of the RGS deletion mutants (Δ*rgs-2*, Δ*rgs-3*, Δ*rgs-4*, Δ*rgs-5*, Δ*rgs-6* and Δ*rgs-7*) possessed significantly taller aerial hyphae than wild type (Figure 2B and Figure S5). In contrast, mutants lacking the *gna-1* or *gna-3* genes had significantly shorter aerial hyphae than wild type. However, the absence of a phenotype in any strain expressing a constitutively activated Gα allele prevents proposing a genetic interaction between a Gα gene and any of the six RGS genes during formation of aerial hyphae. More evidence for epistasis was obtained for quantitation of macroconidia production in agar cultures (Figure 2C and Figure S6). In opposition to the results for aerial hyphae height, all seven RGS knockout mutants produced significantly less macroconidia than wild type during growth on agar medium, with effects most severe in Δ*rgs-2* strains (Figure 2C and Figure S6). Of the three Gα deletion mutants, only Δ*gna-2* differed significantly from wild type, and only from one mating type. However, strains expressing activated versions of *gna-1* or *gna-3* produced significantly less macroconidia than wild type, with *gna-3*^Q208L^ being most affected. The *gna-1*^Q204L^ strain only differs significantly from Δ*rgs-2*, suggesting a possible genetic interaction with the other six *rgs* genes. In contrast, *gna-3*^Q208L^ macroconidia production is most similar to that of Δ*rgs-2* mutants, supporting a genetic interaction between *rgs-2* and *gna-3* during control of macroconidia production on solid medium.

We have previously demonstrated that strains lacking *gna-3* inappropriately produce macroconidia in submerged cultures at a relatively low inoculation density of 5 × 10^5^ or 1 × 10^6^ cells/mL [13,53]. In contrast, Δ*gna-1* mutants only produce macroconidia at a higher inoculation density (3 × 10^6^ cells/mL; [53,54]). In the current study, we assessed macroconidiation in submerged cultures inoculated at the low density of 1 × 10^6^ cells/mL. Consistent with our earlier results, Δ*gna-3* mutants produced macroconidia in submerged liquid cultures. No macroconidia were produced in any of the other Gα or *rgs* deletion mutants, or in strains expressing constitutively activated Gα alleles.

### 3.3. rgs-1 and rgs-2 Exhibit Epistatic Interactions with gna-1 or gna-3 during the Sexual Cycle

We analyzed the strains for three major events during sexual development: the production of protoperithecia, perithecia and ascospores. The findings confirmed published results for the three Gα mutants [11,12,13,19,20], with Δ*gna-2* being like wild type, Δ*gna-1* producing abnormal perithecia and no ascospores, and Δ*gna-3* forming fewer protoperithecia and perithecia than wild type (Table 2). Consistent with previous findings, strains carrying the *gna-1*^Q204L^ allele produced fewer perithecia and ascospores than wild type [11,19], while *gna-2*^Q205L^ strains were normal [12]. In this study, we also observed that *gna-3*^Q208L^ strains did not produce protoperithecia, perithecia or ascospores (Table 2). The female fertility of Δ*rgs-3*, Δ*rgs-4*, Δ*rgs-5*, Δ*rgs-6* and Δ*rgs-7* strains was similar to wild type. In contrast, Δ*rgs-1* mutants did not produce protoperithecia, perithecia or ascospores, while Δ*rgs-2* mutants formed protoperithecia, but reduced numbers of perithecia and ascospores (Table 2). From the phenotypes, it is plausible that *gna-3* shares an epistatic relationship with *rgs-1* during sexual development, in that Δ*rgs-1* and *gna-3*^Q208L^ strains share the same severe sexual cycle phenotypes. In contrast, *gna-1*^Q204L^ strains are most similar to Δ*rgs-2* mutants, consistent with an epistatic relationship between the two genes (Table 2). Thus, it appears that the genetic interactions with *gna-1* and *gna-3* are switched for *rgs-1* and *rgs-2* during asexual vs. sexual development.

### 3.4. Five of the RGS Gene Deletion Mutants Have Phenotypes during Growth on Cellulose and/or in Extracellular Cellulase Activity

The analysis presented above suggests that RGS proteins work in concert with Gα subunits to regulate some aspects of growth and development on medium with sucrose as the carbon source. We next asked whether additional roles for RGS proteins might be observed during growth with cellulose as the sole carbon source. Like other filamentous fungi, *N. crassa* degrades lignocellulosic biomass to generate soluble sugars to use as carbon sources [55]. Cellulose, the most abundant plant polymer in nature, can be degraded to produce glucose monomers (so-called glucose release activity) by a cocktail of secreted enzymes in *N. crassa* [55]. We previously assessed glucose release cellulase activity in cell-free supernatants from G protein mutants by growing cultures on glucose overnight and then transferring to Avicel (crystalline cellulose) for 3 days prior to collecting supernatants for analysis [21]. The results showed that mutants lacking the Gα genes *gna-1* or *gna-3* or components of the Gβγ dimer (*gnb-1*, *cpc-2* or *gng-1*) do not possess detectable glucose release activity [21]. Of interest, strains expressing the *gna-3*^Q208L^ allele do not produce glucose release activity unless the *gnb-1* gene is deleted; this result suggested that a positive role for GNA-3 in regulating cellulase activity is masked by the presence of the Gβ subunit GNB-1 [21]. In contrast to the results for *gna-3*, constitutive activation of *gna-1* or *gna-2* does not lead to reduced cellulase activity [21].

We began our analysis by assessing relative levels of expression for Gα subunits and the seven *rgs* genes in wild type during growth on sucrose vs. cellulose (Avicel). For this work, we took advantage of a publicly available RNAseq dataset [42] (Figure S7). The results showed that *gna-1* is the most highly expressed of the 10 genes during growth on sucrose. Of interest, mRNA levels of *gna-2*, *rgs-3*, *rgs-4* and *rgs-6* are all significantly higher on Avicel vs. sucrose medium, consistent with possible functions during growth on cellulose.

**Figure 3:**
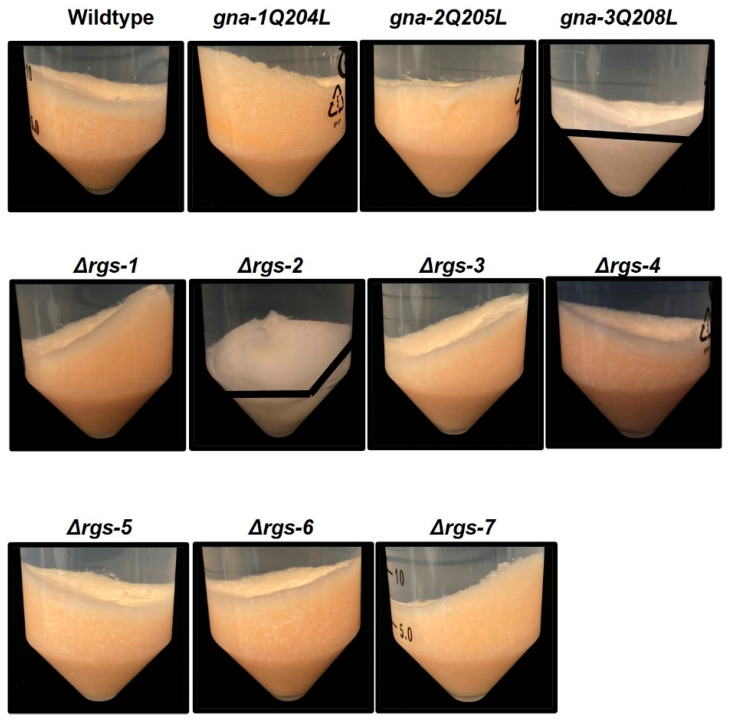
Growth of strains after direct inoculation into Avicel medium. Liquid cultures containing 25mL of VM-Avicel were inoculated with macroconidia at a concentration of 1 × 10^6^ macroconidia/mL and grown with shaking for three days at 25 °C in constant light. After centrifugation, strains that did not completely degrade Avicel into soluble glucose or glucose oligomers have residual Avicel (white powder) remaining in the bottom of the tube. The black line marks the differentiation between the mycelial mat (above the line) and residual Avicel (below the line). Residual Avicel was present in cultures from *gna-3*^Q208L^*,* Δ*rgs-2* and Δ*rgs-7* strains; because the remaining Avicel was dispersed throughout the hyphae in the Δ*rgs-7* strain, it cannot be shown with a black line.

We next explored a role for RGS genes in cellulose degradation using a method we used previously [21]. We inoculated macroconidia into medium with Avicel as the carbon source and then grew the cultures with shaking for 3 days. Because the white Avicel is insoluble, we were able to assess the relative amount of residual Avicel in cultures after centrifugation (Figure 3). After three days, no visible Avicel remained in cultures of the wild-type, *gna-1*^Q204L^, *gna-2*^Q205L^, Δ*rgs-1,* Δ*rgs-3,* Δ*rgs-4,* Δ*rgs-5* and Δ*rgs-6* strains. The results for the *gna-1*^Q204L^ and *gna-2*^Q205L^ strains correlate with our previous observations of normal cellulase activity in these genetic backgrounds [21]. In contrast, *gna-3*^Q208L^, Δ*rgs-2* and Δ*rgs-7* cultures contained some residual Avicel (Avicel was distributed throughout the hyphae in the Δ*rgs-7* mutant). These results suggest that these two RGS genes are required for complete degradation of cellulose into soluble cellodextrins in *N. crassa*. The observation that the *gna-3*^Q208L^ strain is unable to efficiently utilize Avicel is consistent with our previous result demonstrating that this strain lacks detectable glucose release cellulase activity [21].

**Figure 4:**
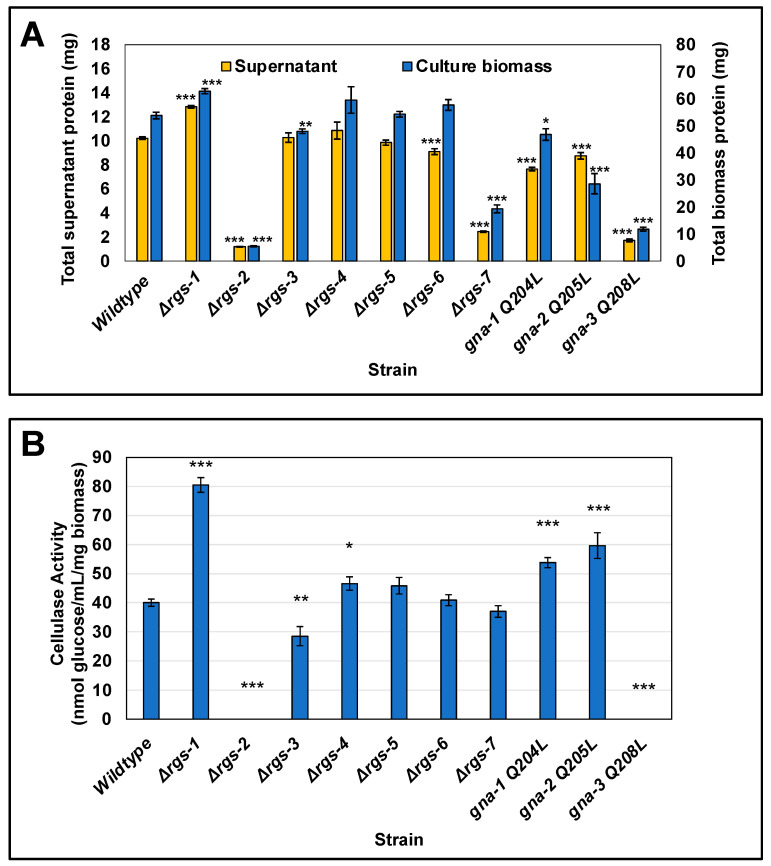
Glucose release cellulase activity and protein amount in culture supernatants and extracted biomass after growth in VM-Avicel for three days. Strains were cultured in VM-Avicel as described in the legend for Figure 3 and grown for three days prior to centrifugation. A sample of each culture supernatant was withdrawn and passed through a 0.45-micron filter. Total protein was extracted from the cell pads of each culture as described in the Materials and Methods. A minimum of three replicates were used for all assays, and errors are expressed as the standard error. Statistical significance relative to wild-type mat A was determined using a two-tailed Student’s *t*-test, and strains with protein levels or cellulase activity significantly different from that of the wild type are indicated as * *p* < 0.05, ** *p* < 0.01, and *** *p* < 0.001. (**A**) Total protein. Protein levels in the cell-free supernatants and the extracted cell pads were determined using the BCA protein assay. The total amount of protein (mg) was calculated using the total volume of the supernatant or the extracted biomass. A summary of all pair-wise statistical comparisons can be found in Figures S8 and S9; (**B**) Glucose release cellulase activity. Glucose release from Avicel activity in the cell-free supernatant was measured as described [21]. Values were normalized to the extracted biomass protein. Units are expressed as nmol glucose/mL culture supernatant/mg biomass protein. A summary of all pair-wise statistical comparisons can be found in Figure S10.

Our second approach to assess possible functions for RGS genes in cellulose degradation was to measure levels of protein and cellulase activity (glucose release from Avicel) in culture supernatants, as well as total protein in the mycelial mat from the seven RGS mutants and wild type grown as described for Figure 3. Levels of supernatant protein were similar to wild type in Δ*rgs-3*, Δ*rgs-4* and Δ*rgs-5* strains, were elevated in Δ*rgs-1* strains and reduced in Δ*rgs-2*, Δ*rgs-6*, Δ*rgs-7*, *gna-1*^Q204L^, *gna-2*^Q205L^ and *gna-3*^Q208L^ strains (Figure 4A and Figure S8). For biomass protein, levels in the Δ*rgs-4*, Δ*rgs-5* and Δ*rgs-6* strains were normal, but were increased in Δ*rgs-1* and *gna-1*^Q204L^ strains and decreased in Δ*rgs-2*, Δ*rgs-3*, Δ*rgs-7*, *gna-2*^Q205L^ and *gna-3*^Q208L^ strains (Figure 4A and Figure S9). The Δ*rgs-1* mutant is the only strain with significantly higher levels for both secreted and biomass protein than wild type (Figure 4A and Figures S8 and S9). In contrast, protein levels in the Δ*rgs-2* and Δ*rgs-7* mutants and the *gna-3*^Q208L^ strain are less than 50% of those observed in wild type (Figure 4A), suggesting a possible regulatory interaction between *gna-3* and *rgs-2* and/or *rgs-7* in control of biomass accumulation and secreted protein levels during growth on cellulose. The observation of reduced biomass protein levels in the *gna-3*^Q208L^, Δ*rgs-2* and Δ*rgs-7* strains is consistent with the presence of residual Avicel in these cultures after three days (Figure 3).

Glucose release cellulase activity in Δ*rgs-5*, Δ*rgs-6* and Δ*rgs-*7 mutants is similar to wild type (Figure 4B and Figure S10). Of note, Δ*rgs-1* mutants have significantly greater activity than wild type (~two-fold increased; Figure 4B and Figure S10). The only other RGS mutant with significantly elevated (*p* < 0.05; Figure S10) activity relative to wild type is Δ*rgs-4*. Elevated cellulase activity is also observed in the *gna-1*^Q204L^ and *gna-2*^Q205L^ strains (Figure 4B and Figure S10), consistent with a possible epistatic relationship between *rgs-1* and/or *rgs-4* and *gna-1* and/or *gna-2*. In contrast, Δ*rgs-3* mutants had significantly reduced activity relative to wild type and cellulase activity could not be detected in Δ*rgs-2* mutants and *gna-3*^Q208L^ strains (Figure 4B). These results suggest possible epistasis between *gna-3* and *rgs-2* and/or *rgs-3*.

**Figure 5:**
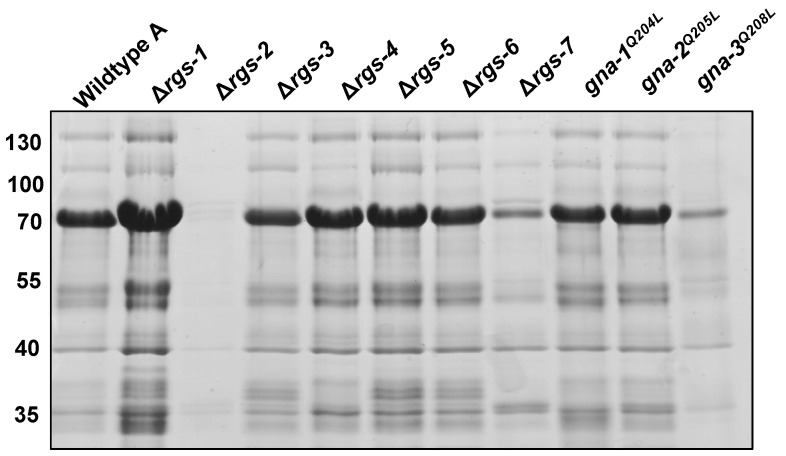
SDS-PAGE analysis of Avicel culture supernatants. One mL of cell-free culture supernatant obtained as described in the legend to Figure 4 was concentrated 5-fold as described [21]. A volume containing 20 μL was subjected to SDS-PAGE using a 10% resolving gel. The positions of the molecular weight markers are shown along the left side of the figure. Note the prominent band at ~70 kDa in the wild type strain, which corresponds to several cellulase enzymes.

We previously showed that cell-free supernatants from G protein mutants with reduced cellulase activity exhibited altered protein banding patterns after SDS-PAGE [21]. By loading equal volumes (not protein) of supernatant on the gel, differences in protein concentration and the protein banding patterns of each strain are highlighted; the SDS-PAGE gel gives a visual representation of the data obtained for supernatant protein concentration (analogous to Figure 4A). In order to investigate these parameters for the RGS mutants, we subjected equal volumes of concentrated cell-free supernatants to SDS-PAGE (Figure 5). As in our earlier study, several major bands were observed in wild type, with the most abundant at ~70 kDa. This band corresponds to the migration position of many cellulase enzymes [56]. We previously demonstrated via single-band proteomics that the most abundant protein in the ~70kDa band in wild type is cellobiohydrolase CBH-1 (NCU07340), at 41% of the total [21]. Other proteins found in lesser amounts in this band were the β-glucosidase gh3-4 (13% ± 0%), the cellobiose dehydrogenase CDH-1 (4% ± 1%), and the cellobiohydrolase gh6-2 (otherwise known as CBH-2; 2% ± 1%) [21]. Inspection of the stained SDS-PAGE gel in the current study revealed that levels of the 70-kDa band were greatly reduced in Δ*rgs-2*, Δ*rgs-7* and *gna-3*^Q208L^ strains (Figure 5), consistent with reduced or non-detectable cellulase activity (Figure 4B). In contrast, levels of the 70kDa band were clearly elevated in the Δ*rgs-1* mutant, in keeping with the significantly higher cellulase activity observed in this strain relative to wild type.

## 4. Discussion

In this study, we characterized phenotypes for mutants lacking the seven RGS genes and compared them to those of strains carrying constitutively activated alleles for each of the three Gα genes (see Table 3 for summary). Our results revealed the strongest support for epistatic relationships between Gα and RGS genes during asexual and sexual development and in regulation of secreted cellulase activity. Strains expressing *gna-1*^Q204L^ or lacking *gna-1*, *rgs-1* or *rgs-3* have significantly slower hyphal growth rates than wild type, suggesting possible genetic interactions between *gna-1* and *rgs-1* and/or *rgs-3* during hyphal growth. RGS-1 and RGS-3 orthologs are required for hyphal growth in other fungi in which they have been studied. For example, RGS-1 orthologs regulate hyphal growth in *A. nidulans, A. fumigatus* and *M. oryzae* [26,57,58]. MoRgs3 is required for spore germination and germ tube growth, along with appressorium formation and virulence in *M. oryzae* [58]. In *A. fumigatus*, mutation of the *rgs-*3 ortholog *rax1* leads to slower growth and decreased conidiation relative to wild type [28]. In *S. cerevisiae* the RGS-3 ortholog Rax1p is necessary for bipolar budding and cell proliferation [59].

We obtained strong evidence for an epistatic relationship between *gna-3* and *rgs-2* during control of macroconidiation. In the case of *gna-1*, the results supported a possible interaction with the other six RGS genes. These findings can be compared with those from *A. nidulans*, where the orthologs of RGS-1 (FlbA) and RGS-2 (RgsA) were predicted to act on those for GNA-1 (FadA) and GNA-3 (GanB), respectively, to control asexual sporulation [57,60]. However, these earlier reports did not include the additional RGS genes later identified in *A. nidulans*. In a study performed using *A. flavus*, mutants lacking all six RGS genes and a strain expressing mutationally activated *fadA* allele were analyzed [31]. The results showed that activation of *fadA* or loss of the RGS genes *rgsA*, *rgsC* or *rgsD* (orthologs of *N. crassa rgs-2*, *rgs-4* and *rgs-5*, respectively) results in increased conidia production relative to wild type, suggesting possible epistasis between *fadA* and these three RGS genes. In contrast, loss of *flbA* (*N. crassa rgs-1* ortholog) led to greatly reduced conidia production [31]. Thus, the GNA-1 ortholog possesses an epistatic interaction with different RGS genes in two different Aspergillus species during control of asexual sporulation.

In previous studies, we have noted an inverse relationship between aerial hyphae height and macroconidia abundance/premature macroconidiation for G protein signaling mutants in *N. crassa*. Examples are strains with constitutive activation of *gna-1* or loss of *gna-1*, *gna-3*, *gnb-1*, *gng-1* or *cr-1* [11,13,17,20,54]. Our new results for the *gna-3*^Q208L^ allele break this paradigm, as the strain has very short aerial hyphae and also produces few macroconidia. We have previously demonstrated an antagonistic role for GNB-1 towards GNA-3 during control of extracellular cellulase activity [21]. Our findings of decreased aerial hyphae and macroconidia formation in *gna-3*^Q208L^ strains may point to a more general role for GNB-1 in antagonizing signaling by GNA-3 in *N. crassa*.

It is noteworthy that RGS-1 appears to regulate GNA-3, while RGS-2 controls GNA-1 during sexual differentiation, which is in opposition to the epistatic relationships noted during asexual growth and development and cellulase activity. In *M. oryzae*, evidence indicates that MoRgs1 and MoRgs4 are required for perithecia formation and mating, and that MoRgs1 acts through regulation of the *N. crassa* GNA-1 ortholog MoMagB [58]. Although the GNA-1 ortholog FadA is required for sexual development (cleistothecia formation and ascospore production) in *A. nidulans* [61], roles for RGS proteins during sexual differentiation have not yet been reported in this species [26].

We previously hypothesized that cycling of GDP and GTP on the Gα subunit GNA-1 is necessary for female fertility in *N. crassa*, since introduction of a GTPase-deficient form of the *gna-1* Gα gene into a mutant lacking the coupled pheromone receptor did not rescue its female sterility [62]. In this study, we demonstrate that loss of *rgs-2* leads to similar sexual cycle defects as constitutive activation of *gna-1*, in keeping with a role for RGS-2 in activating the GTPase activity of GNA-1. Our results for *gna-3*^Q208L^ strain showed that constitutive activation of *gna-3* blocks the very earliest step of female fertility—formation of protoperithecia—consistent with a requirement for GDP/GTP cycling on GNA-3 during sexual development. Thus, the sexual cycle defects newly observed in *gna-3*^Q208L^, Δ*rgs-1* and Δ*rgs-2* mutants in our current study not only support our earlier hypothesis based on GNA-1, but also extend the proposed model to GNA-3.

Our finding of altered cellulase activity in four of the seven RGS knockout mutants further enlarges the number of G protein signaling-related components that are required for cellulase production in *N. crassa*. Activation of *gna-1* or *gna-2* did not lead to higher cellulase activity relative to wild type in our earlier study, which involved growth of strains in glucose overnight followed by transfer to Avicel medium for three days prior to collection [21]. This contrasts with our current results obtained using strains inoculated directly into medium containing Avicel. Under these conditions, we observed highly significant (*p* < 0.001) and elevated cellulase activity in *gna-1*^Q204L^ and *gna-2*^Q205L^ strains and the Δ*rgs-1* mutant. These new results suggest that RGS-1 may function as a GAP for GNA-1 and/or GNA-2 during regulation of cellulase activity. In contrast, the complete absence of detectable cellulase activity in the *gna-3*^Q208L^ strain observed here and in our previous study [21], and for Δ*rgs-2* in this work (*p* < 0.001), supports a model in which RGS-2 acts as a GAP for GNA-3 during regulation of cellulase activity in *N. crassa*.

## Figures and Tables

**Table 1:** Strains used in this study.

Relevant Genotype	Strain Name	Detailed Genotype	NCU Number	Source
Wild type	74-OR23-1A	Wild type, *mat A*	NA ^2^	FGSC2489 ^1^
Wild type	OR8-1a	Wild type *mat a*	NA	FGSC4200
*∆gna-1*	3b10	*∆gna-1:hph, mat a*	NCU06493	Ivey et al., 1999 [18]
*∆gna-2*	gna-2 a	*∆gna-2:hph, mat a*	NCU06729	FGSC12377
*∆gna-3*	31c2	*∆gna-3:hph, mat A*	NCU05206	Kays et al., 2000 [13]
gna-1*	∆1gna-1*	*∆gna-1:hph, gna-1^Q204L^:his-3^+^, mat A*	NA	Collier et al., 2020 [21]
gna-2*	G2-7	*pccg-1::gna-2^Q205L^::his-3^+^, mat A*	NA	Collier et al., 2020 [21]
gna-3*	gna3Q208L	*pccg-1::gna-3^Q208L^::his-3^+^, mat A*	NA	Collier et al., 2020 [21]
*∆rgs-1*	rgs-1-2a	*∆rgs-1::hph, mat a*	NCU08319	This Study
*∆rgs-2*	rgs-2-7a	*∆rgs-2::hph, mat a*	NCU05435	This Study
*∆rgs-3*	rgs-3-2a	*∆rgs-3::hph, mat a*	NCU08343	This Study
*∆rgs-4*	rgs-4-7A	*∆rgs-4::hph, mat A*	NCU03937	This Study
*∆rgs-5*	rgs-5-3	*∆rgs-5::hph, mat a*	NCU09883	This Study
*∆rgs-6*	rgs-6-a	*∆rgs-6::hph, mat a*	NCU03153	FGSC14041
*∆rgs-7*	rgs-7-A	*∆rgs-7::hph, mat A*	NCU09415	FGSC15515
*∆rgs-1, ∆mus-52*	rgs1m7	*∆rgs-1::hph, ∆mus-52::nat, mat a*	NA	This Study
*∆rgs-2, ∆mus-52*	rgs2m2	*∆rgs-2::hph, ∆mus-52::nat, mat a*	NA	This Study
*∆rgs-3, ∆mus-52*	rgs3m1	*∆rgs-3::hph, ∆mus-52::nat, mat a*	NA	This Study
*∆rgs-4, ∆mus-52*	rgs4m5	*∆rgs-4::hph, ∆mus-52::nat, mat a*	NA	This Study
*∆rgs-5, ∆mus-52*	rgs5m4	*∆rgs-5::hph, ∆mus-52::nat, mat A*	NA	This Study
*∆rgs-6, ∆mus-52*	rgs6m1	*∆rgs-6::hph, ∆mus-52::nat, mat a*	NA	This Study
*∆rgs-6, ∆mus-52*	rgs6m5	*∆rgs-6::hph, ∆mus-52::nat, mat a*	NA	This Study
*∆rgs-7, ∆mus-52*	rgs7m1	*∆rgs-7::hph, ∆mus-52::nat, mat a*	NA	This Study
*∆rgs-1, rgs-1+*	rgs1m7-c2	*∆rgs-1::hph, ∆mus-52::nat, pccg-1::rgs-1+::pan-2, mat a*	NA	This Study
*∆rgs-2, rgs-2+*	rgs2m2-c2	*∆rgs-2::hph, ∆mus-52::nat, pccg-1::rgs-2+::pan-2, mat a*	NA	This Study
*∆rgs-3, rgs-3+*	rgs3m1-c5	*∆rgs-3::hph, ∆mus-52::nat, pccg-1::rgs-3+::pan-2, mat a*	NA	This Study
*∆rgs-4, rgs-4+*	rgs4m4-c1	*∆rgs-4::hph, ∆mus-52::nat, pccg-1::rgs-4+::pan-2, mat a*	NA	This Study
*∆rgs-5, rgs-5+*	rgs5m4-c8	*∆rgs-5::hph, ∆mus-52::nat, pccg-1::rgs-5+::pan-2, mat A*	NA	This Study
*∆rgs-6, rgs-6+*	rgs6m1-c3	*∆rgs-6::hph, ∆mus-52::nat, pccg-1::rgs-6+::pan-2, mat a*	NA	This Study
*∆rgs-7, rgs-7+*	rgs7m1-c1	*∆rgs-7::hph, ∆mus-52::nat, pccg-1::rgs-7+::pan-2, mat a*	NA	This Study

^1^ FGSC, Fungal Genetics Stock Center. ^2^ NA, Not applicable.

**Table 2:** Sexual cycle phenotype summary.

Strain Name	Relevant Genotype ^1^	Protoperithecia	Perithecia	Ascospores
74-OR23-1A	Wild type, *mat A*	Normal	Normal	Normal
OR8-1a	Wild type, *mat a*	Normal	Normal	Normal
3b10	*∆gna-1*	Normal	Abnormal	Not Formed
gna-2 a	*∆gna-2*	Normal	Normal	Normal
31c2	*∆gna-3*	Reduced	Reduced	Normal
∆1gna-1*	*gna-1^Q204L^*	Reduced	Reduced	Reduced
G2-7	*gna-2^Q205L^*	Normal	Normal	Normal
gna3Q208L	*gna-3^Q208L^*	Not Formed	Not Formed	Not Formed
rgs-1-2a	*∆rgs-1*	Not Formed	Not Formed	Not Formed
rgs-2-7a	*∆rgs-2*	Normal	Reduced	Reduced
rgs-3-2a	*∆rgs-3*	Normal	Normal	Normal
rgs-4-7A	*∆rgs-4*	Normal	Normal	Normal
rgs-5-3	*∆rgs-5*	Normal	Normal	Normal
rgs-6-a	*∆rgs-6*	Normal	Normal	Normal
rgs-7-A	*∆rgs-7*	Normal	Normal	Normal

^1^ Refer to Table 1 for detailed genotypes.

**Table 3:** Phenotype Summary for RGS Mutants.

Function	RGS Protein Regulation Type/Possible Gα Partner(s) Strongly Supported Partners Are Underlined. —; No Effect due to Mutation of RGS						
	RGS-1	RGS-2	RGS-3	RGS-4	RGS-5	RGS-6	RGS-7
Growth Rate	Positive/GNA-1	—	Positive/GNA-1	—	—	—	—
Aerial Hyphae Height	—	Negative	Negative	Negative	Negative	Negative	Negative
Macroconidia Abundance	Positive/GNA-1	Positive/GNA-3	Positive/GNA-1	Positive/GNA-1	Positive/GNA-1	Positive/GNA-1	Positive/GNA-1
Sexual Development	Positive/GNA-3	Positive/GNA-1	—	—	—	—	—
Avicel Utilization (2 days)	—	Positive/GNA-3	—	Positive/GNA-3	Positive/GNA-3	Positive/GNA-3	Positive/GNA-3
Culture Biomass in Avicel (3 days)	Negative	Positive/GNA-3	Positive/GNA-3	—	—	—	Positive/GNA-3
Glucose Release Cellulase Activity	Negative/GNA-1 GNA-2	Positive/GNA-3	Positive/GNA-3	Negative/GNA-1 GNA-2	—	—	—

## Data Availability

All data reported in this paper are available in the main text or the Supplementary Materials.

## Supplementary Materials

The following supporting information can be downloaded at: https://www.mdpi.com/article/10.3390/jof8101076/s1, Figure S1: Genotyping of RGS knockout deletion mutants using PCR.; Figure S2: Genotyping of RGS complemented strains using PCR; Figure S3: Representative phenotypes of complemented Δ*rgs* mutants; Figure S4: Significance testing for basal hyphae growth rate measurements; Figure S5: Significance testing for aerial hyphae height measurements; Figure S6: Significance testing for macroconidia measurements; Figure S7: Expression of RGS and Gα subunit genes on Avicel and sucrose; Figure S8: Significance testing for supernatant protein measurements; Figure S9: Significance testing for biomass protein measurements; Figure S10: Significance testing for glucose release cellulase activity measurements; Table S1: Primers used in this study.
