# Supplementary material for: Regulator of G Protein Signaling Proteins Control Growth, Development and Cellulase Production in Neurospora crassa
Source: J Fungi (Basel). 2022 Oct 13;8(10):1076. doi: 10.3390/jof8101076 (PMC9604755; doi:10.3390/jof8101076)
Supplement: Supplementary file 1 [file jof-08-01076-s001.zip › jof-1915673-supplementary.pdf]

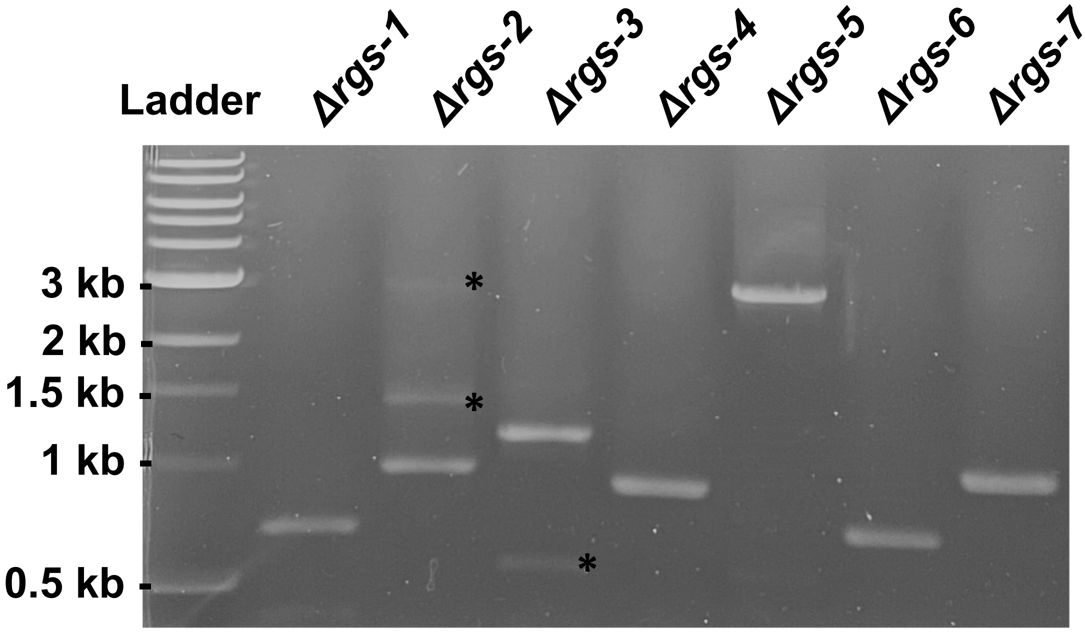


**Figure S1. Genotyping of RGS knockout deletion mutants using PCR.**
The indicated Δrgs strains were checked for proper integration of the gene knockout construct at the correct locus via Polymerase Chain Reactions (PCRs). Genomic DNA was isolated from the indicated genotypes and used in PCRs with the indicated primers (see Table S1). After electrophoresis, agarose gels were stained using ethidium bromide and imaged. The migration position of DNA molecular weight markers (M) in kb is shown to the right of each gel image. The asterisks denote background bands between 0.5 and 3.0 kb in the various lanes.

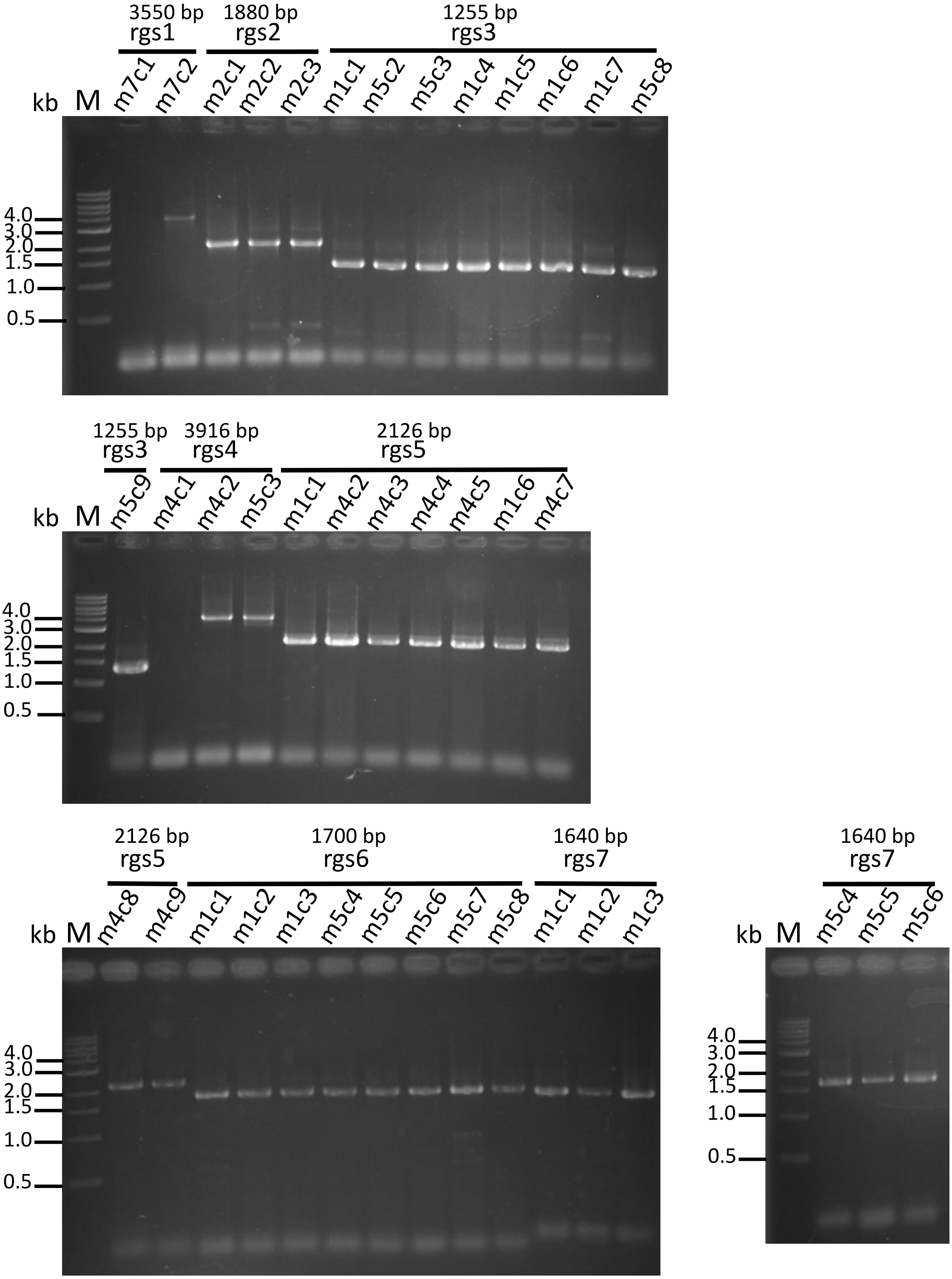


**Figure S2. Genotyping of RGS complemented strains using PCR.**
Δ*rgs* strains were complemented *in trans* by targeting DNA to the *pan-2* locus (see Materials and Methods for details). Strain genotypes are listed in Table 1. Transformants were checked for integration of the DNA construct at the correct locus via Polymerase Chain Reactions (PCRs). Genomic DNA was isolated from the indicated genotypes and used in PCRs with the indicated primers (See Table S1). After electrophoresis, agarose gels were stained using ethidium bromide and imaged. The deleted RGS gene in each group of strains and the predicted size of the PCR fragment after DNA integration are shown above the bar in each panel. The migration position of DNA molecular weight markers (M) in kb is shown to the left of each gel image.

A

B

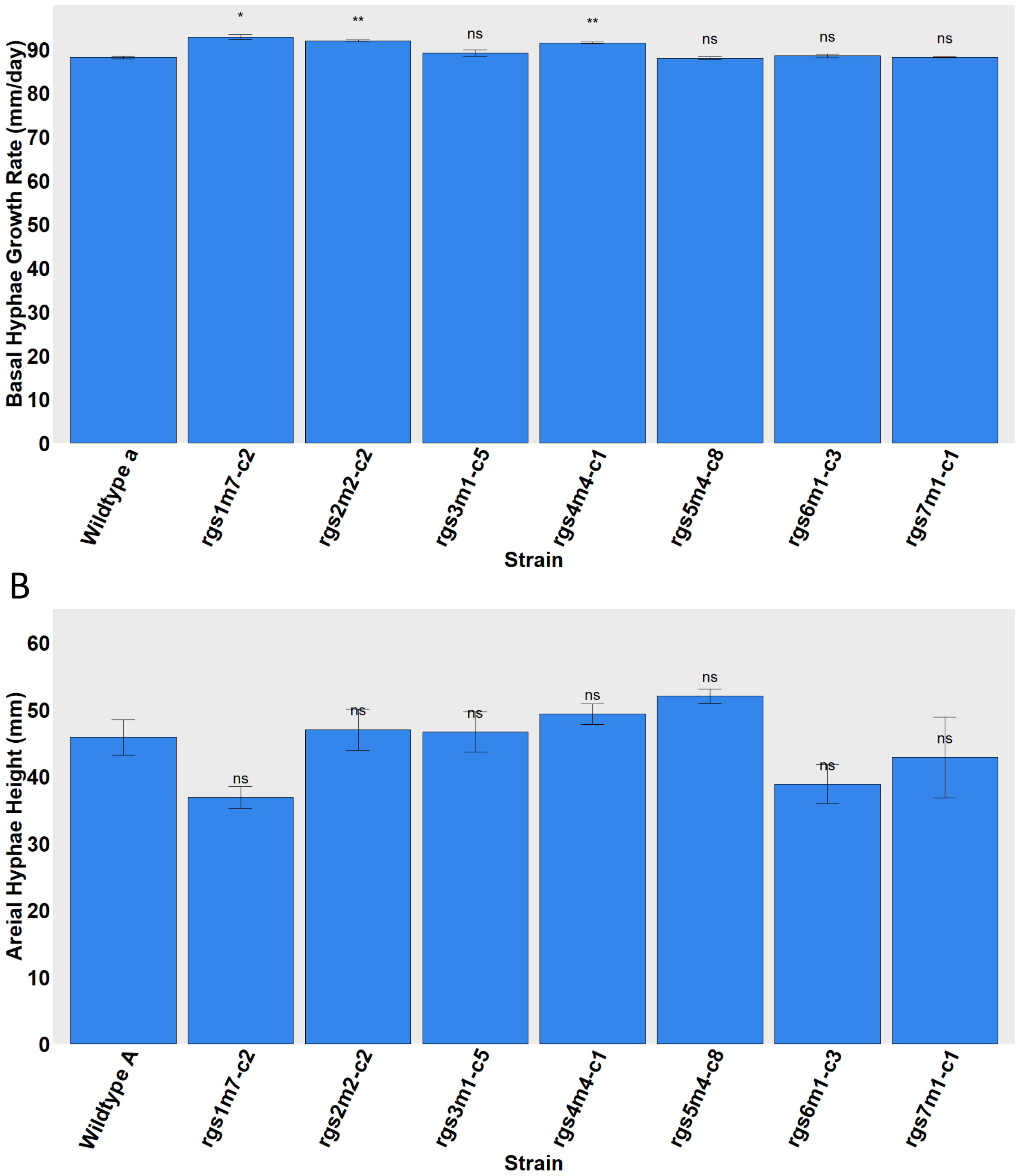


**Figure S3. Representative phenotypes of complemented ∆*rgs* mutants.** Wild type and each of the indicated ∆*rgs* mutants expressing a wild-type copy of the same *rgs* gene targeted to the *pan-2* locus were examined for **(A)** basal hyphae growth rate (4 replicates) and **(B)** aerial hyphae height (12 replicates). The p-value significance levels relative to wild type are denoted as * $p < 0.05$; ** $p<0.01$, and *** $p< 0.001$. See the Methods for details.

| Genotype | Δ*gna-1* | gna-1$^{Q204L}$ | Δ*gna-2* | gna-2$^{Q205L}$ | Δ*gna-3* | gna-3$^{Q208L}$ | Δ*rgs-1* | Δ*rgs-2* | Δ*rgs-3* | Δ*rgs-4* | Δ*rgs-5* | Δ*rgs-6* | Δ*rgs-7* | Wildtype a |
|---|---|---|---|---|---|---|---|---|---|---|---|---|---|---|
| gna-1$^{Q204L}$ | 0.000116 | | | | | | | | | | | | | |
| Δ*gna-2* | 1.76E-06 | 6.31E-05 | | | | | | | | | | | | |
| gna-2$^{Q205L}$ | 4.53E-05 | 0.000427 | 1 | | | | | | | | | | | |
| Δ*gna-3* | 0.006456 | 0.004286 | 3.66E-05 | 0.001047 | | | | | | | | | | |
| gna-3$^{Q208L}$ | 0.004286 | 0.039263 | 0.187105 | 0.187105 | 0.032087 | | | | | | | | | |
| Δ*rgs-1* | 0.06662 | 0.00284 | 8.08E-05 | 0.002434 | 0.068982 | 0.036171 | | | | | | | | |
| Δ*rgs-2* | 2.18E-05 | 0.000126 | 0.06662 | 0.107499 | 0.000338 | 0.011974 | 0.00051 | | | | | | | |
| Δ*rgs-3* | 0.005652 | 1 | 0.004752 | 0.005677 | 0.06068 | 0.052762 | 0.039263 | 0.000427 | | | | | | |
| Δ*rgs-4* | 1.67E-05 | 0.000121 | 0.176224 | 0.245689 | 0.000289 | 0.021053 | 0.000453 | 1 | 0.000645 | | | | | |
| Δ*rgs-5* | 3.1E-06 | 4.21E-05 | 0.197642 | 0.342515 | 7.61E-05 | 0.036171 | 0.000152 | 1 | 0.001216 | 1 | | | | |
| Δ*rgs-6* | 1.55E-05 | 0.000348 | 0.200961 | 0.55104 | 3.13E-07 | 0.187105 | 1.99E-08 | 0.877653 | 0.012275 | 1 | 1 | | | |
| Δ*rgs-7* | 1.06E-06 | 5.99E-05 | 0.245801 | 0.605542 | 1.92E-07 | 0.145116 | 2.36E-07 | 0.605542 | 0.007774 | 1 | 1 | 1 | | |
| Wildtype a | 1.69E-06 | 9.18E-05 | 0.605542 | 1 | 1.79E-07 | 0.187105 | 1.53E-07 | 0.348482 | 0.009882 | 0.915993 | 1 | 0.608872 | 1 | |
| Wildtype A | 4.3E-07 | 2.12E-05 | 0.235041 | 0.521497 | 2.05E-06 | 0.08652 | 6.04E-06 | 0.608872 | 0.004286 | 1 | 1 | 1 | 1 | 1 |

**Figure S4. Significance testing for basal hyphae growth rate measurements.** Students t-test was performed between each pair of strains. The p-values are indicated on the figure.

| Genotype | Δ*gna-1* | gna-1$^{Q204L}$ | Δ*gna-2* | gna-2$^{Q205L}$ | Δ*gna-3* | gna-3$^{Q208L}$ | Δ*rgs-1* | Δ*rgs-2* | Δ*rgs-3* | Δ*rgs-4* | Δ*rgs-5* | Δ*rgs-6* | Δ*rgs-7* | Wildtype a |
|---|---|---|---|---|---|---|---|---|---|---|---|---|---|---|
| gna-1$^{Q204L}$ | 1.49E-14 | | | | | | | | | | | | | |
| Δ*gna-2* | 1.24E-05 | 1 | | | | | | | | | | | | |
| gna-2$^{Q205L}$ | 2.31E-07 | 0.038887 | 0.067094 | | | | | | | | | | | |
| Δ*gna-3* | 0.001998 | 2.62E-14 | 0.00026 | 3.61E-06 | | | | | | | | | | |
| gna-3$^{Q208L}$ | 6.18E-14 | 1 | 1 | 0.017812 | 8.88E-15 | | | | | | | | | |
| Δ*rgs-1* | 1.31E-14 | 1 | 1 | 0.05062 | 2.5E-12 | 1 | | | | | | | | |
| Δ*rgs-2* | 2.12E-12 | 9.64E-06 | 0.001115 | 1 | 2.13E-09 | 4.79E-06 | 9.54E-06 | | | | | | | |
| Δ*rgs-3* | 1.3E-15 | 3.77E-08 | 0.000189 | 0.940804 | 8.24E-12 | 1.94E-08 | 3.16E-08 | 1 | | | | | | |
| Δ*rgs-4* | 1.44E-11 | 7.7E-07 | 5.16E-06 | 0.025854 | 3.27E-09 | 5.57E-07 | 5.17E-07 | 0.030101 | 0.22546 | | | | | |
| Δ*rgs-5* | 1.97E-14 | 2.95E-08 | 1.24E-05 | 0.067094 | 4.05E-11 | 1.96E-08 | 1.81E-08 | 0.069919 | 0.714093 | 1 | | | | |
| Δ*rgs-6* | 3.61E-19 | 5.62E-13 | 2.1E-06 | 0.001836 | 1.49E-14 | 5.6E-13 | 2.87E-13 | 7.16E-05 | 0.000191 | 1 | 0.162789 | | | |
| Δ*rgs-7* | 9.74E-15 | 1.88E-09 | 7.7E-07 | 0.001115 | 6.8E-11 | 2E-09 | 5.59E-10 | 7.93E-05 | 0.000393 | 1 | 0.125538 | 1 | | |
| Wildtype a | 9.97E-09 | 1 | 1 | 0.101794 | 1.34E-06 | 1 | 1 | 0.00015 | 6.71E-06 | 4.11E-07 | 2.81E-07 | 9.61E-09 | 5.07E-09 | |
| Wildtype A | 1.97E-07 | 1 | 1 | 0.244099 | 8.2E-06 | 1 | 1 | 0.001825 | 0.000161 | 3.93E-06 | 6.72E-06 | 4.78E-07 | 1.85E-07 | 1 |

**Figure S5. Significance testing for aerial hyphae height measurements.** Students t-test was performed between each pair of strains. The p-values are indicated on the figure.

| Genotype | Δ*gna-1* | gna-1$^{Q204L}$ | Δ*gna-2* | gna-2$^{Q205L}$ | Δ*gna-3* | gna-3$^{Q208L}$ | Δ*rgs-1* | Δ*rgs-2* | Δ*rgs-3* | Δ*rgs-4* | Δ*rgs-5* | Δ*rgs-6* | Δ*rgs-7* | Wildtype a |
|---|---|---|---|---|---|---|---|---|---|---|---|---|---|---|
| gna-1$^{Q204L}$ | 7.28E-06 | | | | | | | | | | | | | |
| Δ*gna-2* | 0.153024 | 0.00020027 | | | | | | | | | | | | |
| gna-2$^{Q205L}$ | 1 | 2.65658E-05 | 1 | | | | | | | | | | | |
| Δ*gna-3* | 1 | 2.15756E-06 | 0.007675 | 0.080048 | | | | | | | | | | |
| gna-3$^{Q208L}$ | 6.12E-07 | 4.28188E-05 | 5.38E-07 | 1.1E-07 | 9.82E-07 | | | | | | | | | |
| Δ*rgs-1* | 1.63E-05 | 1 | 0.000344 | 9.93E-05 | 2.83E-06 | 0.000341 | | | | | | | | |
| Δ*rgs-2* | 5.67E-07 | 4.63337E-05 | 4.67E-07 | 8.1E-08 | 9.57E-07 | 0.01771 | 0.000386 | | | | | | | |
| Δ*rgs-3* | 0.000245 | 1 | 0.003385 | 0.00117 | 3.9E-05 | 0.001292 | 1 | 0.001538 | | | | | | |
| Δ*rgs-4* | 2.61E-05 | 1 | 0.000985 | 7.5E-05 | 7.89E-06 | 1.18E-05 | 0.781565 | 1.19E-05 | 1 | | | | | |
| Δ*rgs-5* | 0.002157 | 1 | 0.039544 | 0.010026 | 0.000349 | 0.000809 | 1 | 0.000918 | 1 | 1 | | | | |
| Δ*rgs-6* | 0.000159 | 1 | 0.005131 | 0.000892 | 2.32E-05 | 9.93E-05 | 1 | 0.000109 | 1 | 1 | 1 | | | |
| Δ*rgs-7* | 0.00053 | 0.059469693 | 0.060083 | 0.00208 | 9.75E-05 | 3.65E-06 | 0.024298 | 3.57E-06 | 0.121086 | 0.774291 | 1 | 0.847715 | | |
| Wildtype a | 1 | 6.95784E-06 | 0.161742 | 1 | 1 | 4.56E-07 | 1.89E-05 | 4.1E-07 | 0.000296 | 2.32E-05 | 0.002507 | 0.000179 | 0.000495 | |
| Wildtype A | 1 | 3.23806E-06 | 0.01756 | 0.378159 | 1 | 5.3E-08 | 2.04E-05 | 3.71E-08 | 0.000314 | 5.55E-06 | 0.002114 | 0.000129 | 8.65E-05 | 1 |

**Figure S6. Significance testing for macroconidia measurements.** Students t-test was performed between each pair of strains. The p-values are indicated on the figure.

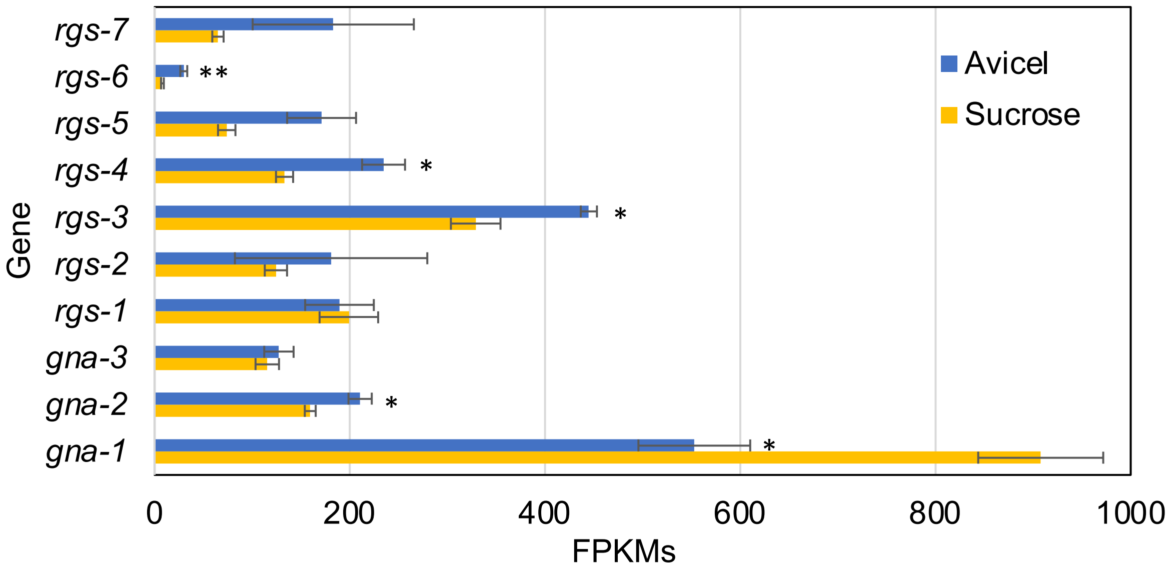


**Figure S7. Expression of RGS and Gα subunit genes on Avicel and sucrose.** RNAseq data were obtained as described in the Materials and Methods for wild type cultured for 16 hours in VM-sucrose liquid medium followed by four more hours in VM-sucrose (yellow bars) or four hours after transfer to VM with Avicel (crystalline cellulose) as the carbon source (blue bars). FPKMs; Fragments per kilobase of transcript per million mapped reads. Students t-test was performed, comparing the gene expression on VM-Avicel to expression of the same gene on VM-sucrose medium. p-value thresholds are indicated as * $p<0.05$, ** $p<0.01$, and *** $p<0.001$.

| Genotype | gna-1$^{Q204L}$ | gna-2$^{Q205L}$ | gna-3$^{Q208L}$ | *Δ rgs-1* | *Δ rgs-2* | *Δ rgs-3* | *Δ rgs-4* | *Δ rgs-5* | *Δ rgs-6* | *Δ rgs-7* |
|---|---|---|---|---|---|---|---|---|---|---|
| gna-2$^{Q205L}$ | 0.006726 | | | | | | | | | |
| gna-3$^{Q208L}$ | 1.93E-15 | 5.4E-08 | | | | | | | | |
| *Δ rgs-1* | 1.9E-14 | 2.98E-06 | 4.63E-21 | | | | | | | |
| *Δ rgs-2* | 3.65E-11 | 9.28E-07 | 0.00208 | 1.59E-14 | | | | | | |
| *Δ rgs-3* | 9.41E-05 | 0.007564 | 3.45E-09 | 0.000134 | 1.29E-08 | | | | | |
| *Δ rgs-4* | 0.001824 | 0.01977 | 8.52E-07 | 0.023674 | 8.04E-07 | 0.485718 | | | | |
| *Δ rgs-5* | 5.6E-07 | 0.008626 | 1.09E-13 | 2.94E-08 | 1.05E-10 | 0.371246 | 0.208477 | | | |
| *Δ rgs-6* | 0.000204 | 0.362165 | 7.4E-12 | 2.12E-08 | 7.47E-10 | 0.025444 | 0.041954 | 0.03377 | | |
| *Δ rgs-7* | 8.32E-12 | 1.27E-06 | 0.000156 | 1.45E-17 | 7.36E-10 | 3.03E-08 | 2.2E-06 | 7.55E-11 | 1.06E-09 | |
| Wildtype A | 5.76E-10 | 0.001735 | 3.54E-19 | 9.75E-12 | 4.06E-14 | 0.917262 | 0.406085 | 0.139249 | 0.001458 | 2.65E-17 |

**Figure S8.** Significance testing for supernatant protein measurements. Students t-test was performed between each pair of strains. The p-values are indicated on the figure.

| Genotype | gna-1$^{Q204L}$ | gna-2$^{Q205L}$ | gna-3$^{Q208L}$ | *Δ rgs-1* | *Δ rgs-2* | *Δ rgs-3* | *Δ rgs-4* | *Δ rgs-5* | *Δ rgs-6* | *Δ rgs-7* |
|---|---|---|---|---|---|---|---|---|---|---|
| gna-2$^{Q205L}$ | 0.001325 | | | | | | | | | |
| gna-3$^{Q208L}$ | 3.77E-08 | 0.002576 | | | | | | | | |
| *Δ rgs-1* | 3E-05 | 2.21E-05 | 2.97E-17 | | | | | | | |
| *Δ rgs-2* | 4.57E-08 | 0.000435 | 8.93E-06 | 5.17E-13 | | | | | | |
| *Δ rgs-3* | 0.605095 | 0.001068 | 4.9E-16 | 2.36E-09 | 1.38E-12 | | | | | |
| *Δ rgs-4* | 0.036335 | 0.000172 | 8.59E-06 | 0.533969 | 4.05E-06 | 0.047234 | | | | |
| *Δ rgs-5* | 0.008827 | 0.000154 | 5.24E-15 | 1.44E-05 | 8.5E-12 | 0.000229 | 0.321737 | | | |
| *Δ rgs-6* | 0.001847 | 2.87E-05 | 9.28E-10 | 0.038963 | 3.41E-09 | 0.000958 | 0.731138 | 0.153679 | | |
| *Δ rgs-7* | 4.5E-08 | 0.044657 | 0.000681 | 1.01E-12 | 1.05E-05 | 4.63E-10 | 1.99E-05 | 9.52E-12 | 1.52E-10 | |
| Wildtype A | 0.014641 | 0.000153 | 4.78E-13 | 3.58E-05 | 7.72E-11 | 0.00167 | 0.283828 | 0.7601 | 0.118498 | 7.82E-12 |

**Figure S9.** Significance testing for biomass protein measurements. Students t-test was performed between each pair of strains. The p-values are indicated on the figure.

| Genotype | gna-1$^{Q204L}$ | gna-2$^{Q205L}$ | gna-3$^{Q208L}$ | *Δ rgs-1* | *Δ rgs-2* | *Δ rgs-3* | *Δ rgs-4* | *Δ rgs-5* | *Δ rgs-6* | *Δ rgs-7* |
|---|---|---|---|---|---|---|---|---|---|---|
| gna-2$^{Q205L}$ | 0.170971 | | | | | | | | | |
| gna-3$^{Q208L}$ | 1.22E-09 | 3.56E-06 | | | | | | | | |
| *Δ rgs-1* | 4.7E-07 | 0.003976 | 1.06E-09 | | | | | | | |
| *Δ rgs-2* | 1.22E-09 | 3.56E-06 | 1 | 1.06E-09 | | | | | | |
| *Δ rgs-3* | 1.67E-05 | 7.21E-05 | 2.37E-05 | 2.11E-09 | 2.37E-05 | | | | | |
| *Δ rgs-4* | 0.026283 | 0.018396 | 4.29E-08 | 3.7E-08 | 4.29E-08 | 0.000451 | | | | |
| *Δ rgs-5* | 0.039258 | 0.017172 | 3.01E-07 | 1.69E-07 | 3.01E-07 | 0.001151 | 0.859775 | | | |
| *Δ rgs-6* | 0.000144 | 0.002841 | 2.57E-08 | 2.82E-09 | 2.57E-08 | 0.005954 | 0.080744 | 0.180033 | | |
| *Δ rgs-7* | 6.71E-05 | 0.000947 | 3.13E-07 | 1.46E-09 | 3.13E-07 | 0.04536 | 0.014558 | 0.040596 | 0.267656 | |
| Wildtype A | 2.32E-05 | 0.002333 | 5.01E-09 | 3.85E-09 | 5.01E-09 | 0.007675 | 0.03726 | 0.108859 | 0.753789 | 0.35042 |

**Figure S10.** Significance testing for glucose release cellulase activity measurements. Students t-test was performed between each pair of strains. The p-values are indicated on the figure.

**Table S1: Primers used in this study**

| Primer name | Gene target | Use/Genomic DNA Template | Sequence (5’-3’)[1] |
|---|---|---|---|
| Rgs1FWDiagnostic | *rgs-1* | Gene knockout validation | CCCGGCTCTTTGTCAGGCCC |
| Rgs2FWDiagnostic | *rgs-2* | “ | CCACCACACCACAACACAACACG |
| Rgs3FWDiagnostic | *rgs-3* | “ | TACTCGCGGGCGACAACGTC |
| Rgs4FWDiagnostic | *rgs-4* | “ | GCCCTGCGTGTACAAAACGAGGA |
| Rgs5FWDiagnostic | *rgs-5* | “ | GGATGTCACCCCCGCAAAGCA |
| Rgs6FWDiagnostic | *rgs-6* | “ | AGAGCGGACTTGGAGAGCCA |
| Rgs7FWDiagnostic | *rgs-7* | “ | AGCGGCCCAAACATACGCCG |
| HphBeginREV | *hph* | “ | TGTGTAGAAGTACTCGCCGATAGTG |
| RGS1CompFOR | *rgs-1* | Complementation construct preparation/ Complemented strain validation | ttcacaacccctcacatcaaccaaaATGTCACACGAGGGCGTGGTCCATC |
| RGS2CompFOR | *rgs-2* | “ | ttcacaacccctcacatcaaccaaaATGCCACCGGCGCGGCTACGAAC |
| RGS3CompFOR | *rgs-3* | “ | ttcacaacccctcacatcaaccaaaATGACGGAGCCCATAGCACACACGC |
| RGS4CompFOR | *rgs-4* | “ | TTCACAACCCCTCACATCAACCAAAATGGCAATGGTTCTTCAAGGAAGAG |
| RGS5CompFOR | *rgs-5* | “ | ttcacaacccctcacatcaaccaaaATGTTTGTCCCACGCCTCCTACAAC |
| RGS6CompFOR | *rgs-6* | “ | ttcacaacccctcacatcaaccaaaATGGGTCTCCTACCTCTCACATACC |
| RGS7CompFOR | *rgs-7* | “ | ttcacaacccctcacatcaaccaaaATGCACTTGCCGGGATGGCTAGTTTG |
| RGS1CompREV | *rgs-1* | Complementation construct preparation | GGATAGGCTTTCCGCCGCCTCCGCCCGTGCGGTTCGACCTGCTCTGGCTG |
| RGS2CompREV | *rgs-2* | “ | GGATAGGCTTTCCGCCGCCTCCGCCGGCCTTGAGCGCAAGAGTGGTGGTTG |
| RGS3CompREV | *rgs-3* | “ | GGATAGGCTTTCCGCCGCCTCCGCCTAGTCGTTTACCCGGTACGAGAATG |
| RGS4CompREV | *rgs-4* | “ | GGATAGGCTTTCCGCCGCCTCCGCCCCCCCCTGGCTGCTGTTTCCTGCCC |
| RGS5CompREV | *rgs-5* | “ | GGATAGGCTTTCCGCCGCCTCCGCCAGCCCGAATAAGCGGCTTCAAGAAG |
| RGS6CompREV | *rgs-6* | “ | GGATAGGCTTTCCGCCGCCTCCGCCAAACAAGTGCCCTCCAGGCACGGCG |
| RGS7CompREV | *rgs-7* | “ | GGATAGGCTTTCCGCCGCCTCCGCCGTCATCACAAACCGTCTCCTTCC |
| 3’UTRpanRVdiag | *pan-2* | Complemented strain validation | CTCAGGCTGCTCAAGAAAGCTACC |

[1] Bases indicated in lower case are homologous to the vector backbone ppan-2V5nat.
